# *N*-(3,5-Dichloro­phen­yl)-2,4-dimethyl­benzene­sulfonamide

**DOI:** 10.1107/S1600536810013504

**Published:** 2010-04-17

**Authors:** P. G. Nirmala, B. Thimme Gowda, Sabine Foro, Hartmut Fuess

**Affiliations:** a Department of Chemistry, Mangalore University, Mangalagangotri 574 199, Mangalore, India; b Institute of Materials Science, Darmstadt University of Technology, Petersenstrasse 23, D-64287 Darmstadt, Germany

## Abstract

In the crystal structure of the title compound, C_14_H_13_Cl_2_NO_2_S, the conformation of the N—C bond in the C—SO_2_—NH—C segment has *gauche* torsions with respect to the S=O bonds. The mol­ecule is bent at the N atom, with an C—SO_2_—NH—C torsion angle of −54.9 (3)°. The two benzene rings are tilted relative to each other by 82.3 (2)°. The mol­ecules are linked into centrosymmetric *R*_2_^2^(8) motifs by N—H⋯O hydrogen bonds and C—H⋯π inter­actions along [100].

## Related literature

For the preparation of the compound, see: Savitha & Gowda (2006 ▶). For our study of the effect of substituents on the structures of *N*-(ar­yl)aryl­sulfonamides, see: Gowda *et al.* (2008 ▶, 2009**a* ▶,b* ▶). For related structures, see: Gelbrich *et al.* (2007 ▶); Perlovich *et al.* (2006 ▶). For hydrogen-bond motifs, see: Bernstein *et al.* (1995 ▶).
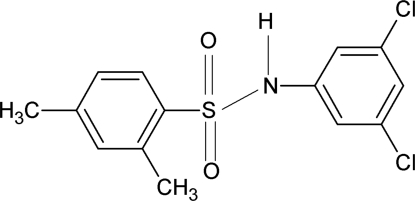


## Experimental

### 

#### Crystal data

C_14_H_13_Cl_2_NO_2_S
*M* *_r_* = 330.21
Monoclinic, 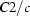

*a* = 23.085 (3) Å
*b* = 8.113 (2) Å
*c* = 16.503 (3) Å
β = 102.03 (2)°
*V* = 3022.9 (10) Å^3^
*Z* = 8
Cu *K*α radiation
μ = 5.16 mm^−1^
*T* = 299 K
0.55 × 0.45 × 0.38 mm

#### Data collection

Enraf–Nonius CAD-4 diffractometer
Absorption correction: ψ scan (North *et al.*, 1968 ▶) *T*_min_ = 0.164, *T*_max_ = 0.245
7561 measured reflections
2690 independent reflections
2340 reflections with *I* > 2σ(*I*)
*R*_int_ = 0.089
3 standard reflections every 120 min  intensity decay: 1.0%

#### Refinement

*R*[*F* ^2^ > 2σ(*F* ^2^)] = 0.053
*wR*(*F* ^2^) = 0.189
*S* = 1.14
2690 reflections
187 parameters
H atoms treated by a mixture of independent and constrained refinement
Δρ_max_ = 0.56 e Å^−3^
Δρ_min_ = −0.46 e Å^−3^

### 

Data collection: *CAD-4-PC* (Enraf–Nonius, 1996 ▶); cell refinement: *CAD-4-PC*; data reduction: *REDU4* (Stoe & Cie, 1987 ▶); program(s) used to solve structure: *SHELXS97* (Sheldrick, 2008 ▶); program(s) used to refine structure: *SHELXL97* (Sheldrick, 2008 ▶); molecular graphics: *PLATON* (Spek, 2009 ▶); software used to prepare material for publication: *SHELXL97*.

## Supplementary Material

Crystal structure: contains datablocks I, global. DOI: 10.1107/S1600536810013504/bx2275sup1.cif

Structure factors: contains datablocks I. DOI: 10.1107/S1600536810013504/bx2275Isup2.hkl

Additional supplementary materials:  crystallographic information; 3D view; checkCIF report

## Figures and Tables

**Table 1:** Hydrogen-bond geometry (Å, °) *Cg*1 is the centroid of the C1–C6 ring.

*D*—H⋯*A*	*D*—H	H⋯*A*	*D*⋯*A*	*D*—H⋯*A*
N1—H1*N*⋯O1^i^	0.86 (4)	2.05 (5)	2.900 (4)	168 (4)
C10—H10⋯*Cg*1^ii^	0.93	2.92	3.834 (4)	168

Symmetry codes: (i) 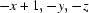; (ii) 
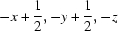
.

**Fig. 1.**
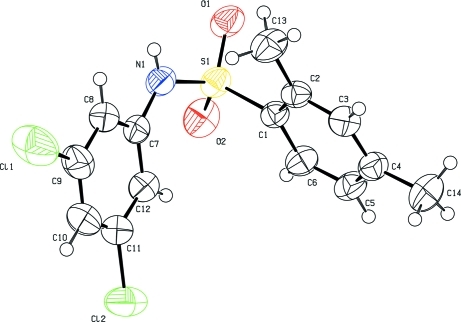
Molecular structure of the title compound, showing the atom labelling scheme. The displacement ellipsoids are drawn at the 50% probability level. The H atoms are represented as small spheres of arbitrary radii.

**Fig. 2.**
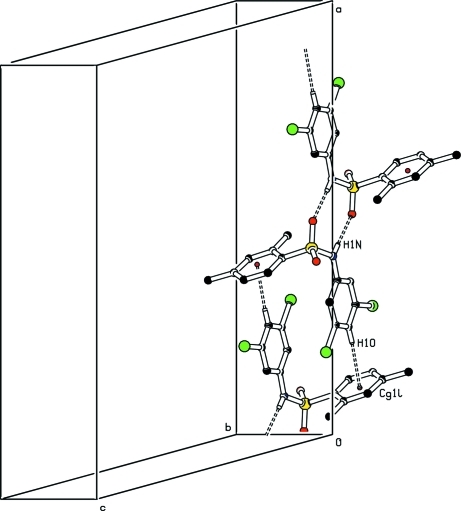
Molecular packing of the title compound with hydrogen bonding shown as dashed lines.

## supplementary crystallographic information

### Comment

As part of a study of substituent effects on the structures of *N*-(aryl)arylsulfonamides (Gowda *et al.*, 2008; 2009*a,b*), we report here  the  crystal structure of the title compound (I) , (Fig. 1). The conformation of the N—C bond in the C—SO_2_—NH—C segment of the structure has gauche torsions with respect to the S═O bonds. The molecule is bent at the *N* atom with the C1—SO_2_—NH—C7 torsion angle of -54.9 (3)°, compared to the values of 46.1 (3)° (glide image of molecule 1) and 47.7 (3)° (molecule 2) in the two independent molecules of 2,4-dimethyl-*N*-(phenyl)benzenesulfonamide (II) (Gowda *et al.*, 2009*a*), -68.1 (3)° in *N*-(3,5-dichlorophenyl)benzenesulfonamide (III)(Gowda *et al.*, 2008) ; 53.9 (2)° in 2,4-dimethyl-*N*-(3,5-dimethylphenyl)- benzenesulfonamide (IV) (Gowda *et al.*, 2009*b*) and -69.7 (2)° in 2,4-dimethyl-*N*-(3,4-dichlorophenyl)benzenesulfonamide (V) (Gowda *et al.*, 2009*b*).

The two benzene rings in (I) are tilted relative to each other by 82.3 (1)°, compared to the values of 67.5 (1)° (molecule 1) and 72.9 (1)° (molecule 2) in the two independent molecules of (II), 57.0 (1)° in (III), 82.1 (1)° in (IV) and 82.4 (1)° in (V). The other atomic  parameters in (I) are similar to those observed in (II), (III), (IV), (V) and other aryl sulfonamides (Perlovich *et al.*, 2006; Gelbrich *et al.*, 2007) as representative examples.The molecules are linked into centrosymmetric R_2_^2^(8) motifs by N—H···O hydrogen bonds and C—H···π interactions along [1 0 0] (Bernstein *et al.*,1995), Fig. 2.

### Experimental

The solution of 1,3-xylene (1,3-dimethylbenzene) (10 ml) in chloroform (40 ml) was treated dropwise with chlorosulfonic acid (25 ml) at 0 ° C. After the initial evolution of hydrogen chloride subsided, the reaction mixture was brought to room temperature and poured into crushed ice in a beaker. The chloroform layer was separated, washed with cold water and allowed to evaporate slowly. The residual 2,4-dimethylbenzenesulfonylchloride was treated with 3,5-dichloroaniline in the stoichiometric ratio and boiled for ten minutes. The reaction mixture was then cooled to room temperature and added to ice cold water (100 ml). The resultant solid 2,4-dimethyl-*N*-(3,5-dichlorophenyl)benzenesulfonamide was filtered under suction and washed thoroughly with cold water. It was then recrystallized to constant melting point from dilute ethanol. The purity of the compound was checked and characterized by recording its infrared and NMR spectra (Savitha & Gowda, 2006).

The prism like colourless single crystals used in X-ray diffraction studies were grown in ethanolic solution by a slow evaporation at room temperature.

### Refinement

The H atom of the NH group was located in a difference map and its position refined with N—H = 0.86 (4) Å. The other H atoms were positioned with idealized geometry using a riding model with C—H = 0.93–0.96 Å A l l H atoms were refined with isotropic displacement parameters (set to 1.2 times of the *U*_eq_ of the parent atom).

### Crystal data

**Table:**

C_14_H_13_Cl_2_NO_2_S	*F*(000) = 1360
*M**_r_* = 330.21	*D*_x_ = 1.451 Mg m^−^^3^
Monoclinic, *C*2/*c*	Cu *K*α radiation, λ = 1.54180 Å
Hall symbol: -C 2yc	Cell parameters from 25 reflections
*a* = 23.085 (3) Å	θ = 5.5–18.6°
*b* = 8.113 (2) Å	µ = 5.16 mm^−^^1^
*c* = 16.503 (3) Å	*T* = 299 K
β = 102.03 (2)°	Prism, colourless
*V* = 3022.9 (10) Å^3^	0.55 × 0.45 × 0.38 mm
*Z* = 8	

### Data collection

**Table:**

Enraf–Nonius CAD-4 diffractometer	2340 reflections with *I* > 2σ(*I*)
Radiation source: fine-focus sealed tube	*R*_int_ = 0.089
graphite	θ_max_ = 66.9°, θ_min_ = 3.9°
ω/2θ scans	*h* = −15→27
Absorption correction: ψ scan (North *et al.*, 1968)	*k* = −9→9
*T*_min_ = 0.164, *T*_max_ = 0.245	*l* = −19→19
7561 measured reflections	3 standard reflections every 120 min
2690 independent reflections	intensity decay: 1.0%

### Refinement

**Table:**

Refinement on *F*^2^	Secondary atom site location: difference Fourier map
Least-squares matrix: full	Hydrogen site location: inferred from neighbouring sites
*R*[*F*^2^ > 2σ(*F*^2^)] = 0.053	H atoms treated by a mixture of independent and constrained refinement
*wR*(*F*^2^) = 0.189	*w* = 1/[σ^2^(*F*_o_^2^) + (0.1031*P*)^2^ + 3.0862*P*] where *P* = (*F*_o_^2^ + 2*F*_c_^2^)/3
*S* = 1.14	(Δ/σ)_max_ = 0.006
2690 reflections	Δρ_max_ = 0.56 e Å^−^^3^
187 parameters	Δρ_min_ = −0.46 e Å^−^^3^
0 restraints	Extinction correction: *SHELXL97* (Sheldrick, 2008), Fc^*^=kFc[1+0.001xFc^2^λ^3^/sin(2θ)]^-1/4^
Primary atom site location: structure-invariant direct methods	Extinction coefficient: 0.00090 (16)

### Special details

**Table:**

Geometry. All esds (except the esd in the dihedral angle between two l.s. planes) are estimated using the full covariance matrix. The cell esds are taken into account individually in the estimation of esds in distances, angles and torsion angles; correlations between esds in cell parameters are only used when they are defined by crystal symmetry. An approximate (isotropic) treatment of cell esds is used for estimating esds involving l.s. planes.
Refinement. Refinement of *F*^2^ against ALL reflections. The weighted *R*-factor wR and goodness of fit *S* are based on *F*^2^, conventional *R*-factors *R* are based on *F*, with *F* set to zero for negative *F*^2^. The threshold expression of *F*^2^ > σ(*F*^2^) is used only for calculating *R*-factors(gt) etc. and is not relevant to the choice of reflections for refinement. *R*-factors based on *F*^2^ are statistically about twice as large as those based on *F*, and *R*- factors based on ALL data will be even larger.

### Fractional atomic coordinates and isotropic or equivalent isotropic displacement parameters (Å^2^)

**Table:**

	*x*	*y*	*z*	*U*_iso_*/*U*_eq_	
C1	0.43013 (12)	0.1399 (3)	0.15596 (17)	0.0411 (6)	
C2	0.44290 (12)	0.2976 (4)	0.13142 (18)	0.0442 (6)	
C3	0.43210 (14)	0.4268 (4)	0.1793 (2)	0.0509 (7)	
H3	0.4397	0.5330	0.1631	0.061*	
C4	0.41035 (13)	0.4069 (4)	0.2511 (2)	0.0507 (7)	
C5	0.39736 (14)	0.2499 (4)	0.27317 (19)	0.0541 (8)	
H5	0.3822	0.2338	0.3205	0.065*	
C6	0.40659 (14)	0.1169 (4)	0.22618 (19)	0.0505 (7)	
H6	0.3971	0.0114	0.2412	0.061*	
C7	0.35181 (14)	0.0098 (4)	−0.0243 (2)	0.0479 (7)	
C8	0.33700 (14)	0.0724 (4)	−0.1038 (2)	0.0537 (8)	
H8	0.3663	0.0948	−0.1332	0.064*	
C9	0.27851 (16)	0.1011 (5)	−0.1390 (2)	0.0653 (9)	
C10	0.23406 (16)	0.0720 (6)	−0.0964 (3)	0.0728 (11)	
H10	0.1946	0.0926	−0.1205	0.087*	
C11	0.24997 (17)	0.0121 (5)	−0.0178 (3)	0.0658 (10)	
C12	0.30817 (16)	−0.0218 (5)	0.0207 (2)	0.0594 (9)	
H12	0.3176	−0.0639	0.0742	0.071*	
C13	0.46692 (19)	0.3326 (5)	0.0555 (2)	0.0657 (10)	
H13A	0.4371	0.3088	0.0070	0.079*	
H13B	0.5010	0.2648	0.0557	0.079*	
H13C	0.4780	0.4466	0.0551	0.079*	
C14	0.4006 (2)	0.5542 (5)	0.3010 (3)	0.0769 (12)	
H14A	0.3644	0.6078	0.2751	0.092*	
H14B	0.4331	0.6296	0.3040	0.092*	
H14C	0.3982	0.5200	0.3558	0.092*	
Cl1	0.26077 (5)	0.1724 (2)	−0.23956 (8)	0.1056 (6)	
Cl2	0.19530 (5)	−0.0269 (2)	0.03757 (9)	0.1007 (5)	
N1	0.41245 (12)	−0.0223 (4)	0.00734 (18)	0.0542 (7)	
H1N	0.4333 (19)	0.011 (5)	−0.027 (3)	0.065*	
O1	0.50684 (10)	−0.0457 (3)	0.10170 (15)	0.0615 (7)	
O2	0.41982 (13)	−0.1739 (3)	0.13907 (17)	0.0680 (7)	
S1	0.44494 (3)	−0.03942 (9)	0.10412 (5)	0.0485 (3)	

### Atomic displacement parameters (Å^2^)

**Table:**

	*U*^11^	*U*^22^	*U*^33^	*U*^12^	*U*^13^	*U*^23^
C1	0.0405 (13)	0.0437 (14)	0.0396 (13)	0.0051 (11)	0.0094 (11)	0.0025 (11)
C2	0.0423 (14)	0.0489 (15)	0.0427 (14)	0.0023 (12)	0.0115 (11)	0.0066 (12)
C3	0.0515 (16)	0.0429 (15)	0.0588 (18)	0.0019 (13)	0.0128 (14)	0.0043 (13)
C4	0.0469 (15)	0.0571 (18)	0.0489 (16)	0.0066 (13)	0.0117 (13)	−0.0040 (14)
C5	0.0572 (17)	0.0655 (19)	0.0440 (15)	0.0038 (15)	0.0207 (13)	0.0031 (14)
C6	0.0584 (17)	0.0503 (16)	0.0460 (16)	0.0012 (13)	0.0181 (13)	0.0088 (13)
C7	0.0446 (15)	0.0497 (15)	0.0491 (16)	0.0012 (12)	0.0092 (12)	−0.0122 (13)
C8	0.0481 (16)	0.0595 (18)	0.0534 (18)	0.0003 (14)	0.0105 (13)	−0.0058 (14)
C9	0.0530 (18)	0.079 (2)	0.059 (2)	−0.0018 (17)	−0.0004 (15)	−0.0007 (18)
C10	0.0436 (17)	0.094 (3)	0.076 (3)	0.0021 (18)	0.0022 (17)	−0.010 (2)
C11	0.0499 (18)	0.081 (2)	0.069 (2)	−0.0055 (17)	0.0176 (17)	−0.0172 (19)
C12	0.0541 (18)	0.072 (2)	0.0534 (19)	−0.0005 (15)	0.0141 (15)	−0.0095 (15)
C13	0.085 (2)	0.062 (2)	0.059 (2)	−0.0049 (18)	0.0346 (19)	0.0102 (16)
C14	0.090 (3)	0.071 (3)	0.075 (3)	0.010 (2)	0.028 (2)	−0.0170 (19)
Cl1	0.0679 (7)	0.1625 (14)	0.0765 (7)	−0.0020 (7)	−0.0073 (5)	0.0331 (8)
Cl2	0.0616 (6)	0.1549 (13)	0.0940 (9)	−0.0108 (6)	0.0353 (6)	−0.0121 (8)
N1	0.0481 (14)	0.0703 (18)	0.0441 (14)	0.0076 (12)	0.0096 (12)	−0.0056 (12)
O1	0.0525 (13)	0.0771 (17)	0.0535 (13)	0.0247 (11)	0.0077 (10)	−0.0028 (11)
O2	0.0908 (18)	0.0436 (12)	0.0717 (16)	0.0060 (12)	0.0214 (14)	0.0064 (11)
S1	0.0521 (5)	0.0462 (5)	0.0475 (5)	0.0114 (3)	0.0106 (3)	0.0007 (3)

### Geometric parameters (Å, °)

**Table:**

C1—C6	1.391 (4)	C9—C10	1.379 (6)
C1—C2	1.392 (4)	C9—Cl1	1.725 (4)
C1—S1	1.757 (3)	C10—C11	1.362 (6)
C2—C3	1.366 (4)	C10—H10	0.9300
C2—C13	1.500 (4)	C11—C12	1.389 (5)
C3—C4	1.389 (5)	C11—Cl2	1.735 (4)
C3—H3	0.9300	C12—H12	0.9300
C4—C5	1.375 (5)	C13—H13A	0.9600
C4—C14	1.495 (5)	C13—H13B	0.9600
C5—C6	1.371 (5)	C13—H13C	0.9600
C5—H5	0.9300	C14—H14A	0.9600
C6—H6	0.9300	C14—H14B	0.9600
C7—C8	1.381 (5)	C14—H14C	0.9600
C7—C12	1.394 (5)	N1—S1	1.623 (3)
C7—N1	1.413 (4)	N1—H1N	0.86 (4)
C8—C9	1.374 (5)	O1—S1	1.439 (2)
C8—H8	0.9300	O2—S1	1.414 (3)
			
C6—C1—C2	120.6 (3)	C9—C10—H10	121.2
C6—C1—S1	116.4 (2)	C10—C11—C12	123.3 (4)
C2—C1—S1	123.0 (2)	C10—C11—Cl2	119.0 (3)
C3—C2—C1	117.5 (3)	C12—C11—Cl2	117.6 (3)
C3—C2—C13	118.8 (3)	C11—C12—C7	117.2 (4)
C1—C2—C13	123.7 (3)	C11—C12—H12	121.4
C2—C3—C4	123.1 (3)	C7—C12—H12	121.4
C2—C3—H3	118.5	C2—C13—H13A	109.5
C4—C3—H3	118.5	C2—C13—H13B	109.5
C5—C4—C3	118.1 (3)	H13A—C13—H13B	109.5
C5—C4—C14	121.9 (3)	C2—C13—H13C	109.5
C3—C4—C14	120.0 (3)	H13A—C13—H13C	109.5
C6—C5—C4	120.7 (3)	H13B—C13—H13C	109.5
C6—C5—H5	119.6	C4—C14—H14A	109.5
C4—C5—H5	119.6	C4—C14—H14B	109.5
C5—C6—C1	120.0 (3)	H14A—C14—H14B	109.5
C5—C6—H6	120.0	C4—C14—H14C	109.5
C1—C6—H6	120.0	H14A—C14—H14C	109.5
C8—C7—C12	120.7 (3)	H14B—C14—H14C	109.5
C8—C7—N1	116.7 (3)	C7—N1—S1	126.8 (2)
C12—C7—N1	122.6 (3)	C7—N1—H1N	110 (3)
C9—C8—C7	119.4 (3)	S1—N1—H1N	118 (3)
C9—C8—H8	120.3	O2—S1—O1	118.57 (16)
C7—C8—H8	120.3	O2—S1—N1	108.85 (17)
C8—C9—C10	121.7 (4)	O1—S1—N1	103.52 (15)
C8—C9—Cl1	118.6 (3)	O2—S1—C1	107.61 (14)
C10—C9—Cl1	119.7 (3)	O1—S1—C1	109.74 (14)
C11—C10—C9	117.7 (3)	N1—S1—C1	108.13 (14)
C11—C10—H10	121.2		
			
C6—C1—C2—C3	0.8 (4)	Cl1—C9—C10—C11	178.5 (3)
S1—C1—C2—C3	−176.7 (2)	C9—C10—C11—C12	−0.5 (7)
C6—C1—C2—C13	−178.5 (3)	C9—C10—C11—Cl2	−179.7 (3)
S1—C1—C2—C13	4.0 (4)	C10—C11—C12—C7	0.7 (6)
C1—C2—C3—C4	1.3 (5)	Cl2—C11—C12—C7	180.0 (3)
C13—C2—C3—C4	−179.5 (3)	C8—C7—C12—C11	0.0 (5)
C2—C3—C4—C5	−2.2 (5)	N1—C7—C12—C11	−178.8 (3)
C2—C3—C4—C14	179.0 (3)	C8—C7—N1—S1	159.3 (3)
C3—C4—C5—C6	1.0 (5)	C12—C7—N1—S1	−21.8 (5)
C14—C4—C5—C6	179.9 (3)	C7—N1—S1—O2	61.7 (3)
C4—C5—C6—C1	0.9 (5)	C7—N1—S1—O1	−171.3 (3)
C2—C1—C6—C5	−1.9 (5)	C7—N1—S1—C1	−54.9 (3)
S1—C1—C6—C5	175.8 (2)	C6—C1—S1—O2	9.6 (3)
C12—C7—C8—C9	−1.0 (5)	C2—C1—S1—O2	−172.8 (3)
N1—C7—C8—C9	177.9 (3)	C6—C1—S1—O1	−120.7 (3)
C7—C8—C9—C10	1.3 (6)	C2—C1—S1—O1	56.9 (3)
C7—C8—C9—Cl1	−177.8 (3)	C6—C1—S1—N1	127.0 (2)
C8—C9—C10—C11	−0.5 (7)	C2—C1—S1—N1	−55.4 (3)

### Hydrogen-bond geometry (Å, °)

**Table:**

Cg1 is the centroid of the C1–C6 ring.

**Table:**

*D*—H···*A*	*D*—H	H···*A*	*D*···*A*	*D*—H···*A*
N1—H1N···O1^i^	0.86 (4)	2.05 (5)	2.900 (4)	168 (4)
C10—H10···Cg1^ii^	0.93	2.92	3.834 (4)	168

Symmetry codes: (i) −*x*+1, −*y*, −*z*; (ii) −*x*+1/2, −*y*+1/2, −*z*.

## References

[bb1] Bernstein, J., Davis, R. E., Shimoni, L. & Chang, N.-L. (1995). *Angew. Chem. Int. Ed. Engl.* **34**, 1555–1573.

[bb2] Enraf–Nonius (1996). *CAD-4-PC* Enraf–Nonius, Delft, The Netherlands.

[bb3] Gelbrich, T., Hursthouse, M. B. & Threlfall, T. L. (2007). *Acta Cryst.* B**63**, 621–632. 10.1107/S010876810701395X 17641433

[bb4] Gowda, B. T., Foro, S., Babitha, K. S. & Fuess, H. (2008). *Acta Cryst.* E**64**, o2190. 10.1107/S1600536808034351 PMC2959669 21581048

[bb5] Gowda, B. T., Foro, S., Nirmala, P. G., Babitha, K. S. & Fuess, H. (2009*a*). *Acta Cryst.* E**65**, o576. 10.1107/S160053680900573X PMC2968648 21582231

[bb6] Gowda, B. T., Foro, S., Nirmala, P. G. & Fuess, H. (2009*b*). *Acta Cryst.* E**65**, o3275. 10.1107/S1600536809050740 PMC2972036 21578969

[bb7] North, A. C. T., Phillips, D. C. & Mathews, F. S. (1968). *Acta Cryst.* A**24**, 351–359.

[bb8] Perlovich, G. L., Tkachev, V. V., Schaper, K.-J. & Raevsky, O. A. (2006). *Acta Cryst.* E**62**, o780–o782.

[bb9] Savitha, M. B. & Gowda, B. T. (2006). *Z. Naturforsch. Teil A*, **60**, 600–606.

[bb10] Sheldrick, G. M. (2008). *Acta Cryst.* A**64**, 112–122. 10.1107/S0108767307043930 18156677

[bb11] Spek, A. L. (2009). *Acta Cryst.* D**65**, 148–155. 10.1107/S090744490804362X PMC2631630 19171970

[bb12] Stoe & Cie (1987). *REDU4* Stoe & Cie GmbH, Darmstadt, Germany.

